# Flexural Behavior of GFRP Tubes Filled with Magnetically Driven Concrete

**DOI:** 10.3390/ma11010092

**Published:** 2018-01-08

**Authors:** Fang Xie, Ju Chen, Xinlong Dong, Bing Feng

**Affiliations:** 1 Faculty of Mechanical Engineering & Mechanics, Ningbo University, Ningbo 315211, China; xiefangusx@163.com (F.X.); XinlongDong_2000@163.com (X.D.); 2 Department of Civil Engineering, Shaoxing University, Shaoxing 312000, China; 3 Department of Civil Engineering, Zhejiang University, Hangzhou 310058, China; 4 Shaoxing Electric Power Bureau, Shaoxing 312000, China; zepdifb@163.com

**Keywords:** bending test, elastic stiffness, GFRP tube, magnetically driven concrete

## Abstract

Experimental investigation of GFRP (glass fiber reinforced polymer) tubes that were filled with magnetically driven concrete was carried out to study the flexural behavior of specimens under bending. Specimens having different cross section and lengths were tested. The test specimens were fabricated by filling magnetically driven concrete into the GFRP tubes and the concrete was vibrated using magnetic force. Specimens vibrated using vibrating tube were also tested for comparison. In addition, specimens having steel reinforcing bars and GFRP bars were both tested to study the effect of reinforcing bars on the magnetically driven concrete. The load-displacement curves, load-strain curves, failure mode, and ultimate strengths of test specimens were obtained. Design methods for the flexural stiffness of test specimens were also discussed in this study.

## 1. Introduction

Since the application of concrete from the nineteenth century, it is always vibrated by mechanical force. Recently, the magnetically driven concrete was proposed by Chen et al. [1]. In the magnetically driven concrete, steel slag was used as coarse and fine aggregate and magnetic field was used to vibrate the concrete. The magnetic vibration method has the advantages of uniform vibration, no space requirement as vibrating tube, no noise, and is controllable. It is shown that the magnetic force is enough to vibrate the concrete for cubic concrete specimens [1]. However, the application of magnetically driven concrete in the structural member level needs to be investigated. Since the steel tube will block the magnetic field [2], the glass fiber reinforced polymer (GFRP) tube was used in this study. The steel reinforcing bars inside the concrete may affect the magnetic field; therefore, the effect of reinforcing bars was investigated by comparing with the counter pairs having GFRP bars.

Due to its excellent performance for high strength, light weight, and corrosion resistance, fiber reinforced polymer (FRP) has promising application in structural engineering. There are many researches towards the GFRP tubes filled with normal concrete under bending. Mirmiran and Shahawy [3] investigated the behavior of concrete columns confined by fiber composites. Mirmiran, et al. [4] conducted experimental investigation on beam-column tests and bending test on concrete-filled GFRP tubes. Davol et al. [5] studied the flexural behavior of circular concrete filled FRP shells. Fam and Rizkalla [6] studied the flexural behavior of concrete-filled fiber-reinforced polymer circular tubes by conducting 20 beams test. Shao [7] investigated the behavior of FRP-concrete beam-columns under cyclic loading. Teng and Lam [8] summarized the previous research and proposed model of fiber reinforced polymer-confined concrete. Aydin and Saribiyik [9] conducted investigation of flexural behaviors of hybrid beams formed with GFRP box section and concrete. Abouzied and Masmoudi [10] studied the behavior of new fully and partially concrete-filled rectangular FRP-tube beams. Muttashar et al. [11] investigated the behavior of hollow pultruded GFRP square beams with different shear span-to-depth ratios. Muttashar et al. [12] investigated the influence of infill concrete strength on the flexural behavior of pultruded GFRP square beams. Muttashar et al. [13] also investigated the flexural behavior of multi-celled GFRP composite beams with concrete infill by experimental investigation. However, those test results are all based on the normal concrete filled in GFRP tubes, and there is lack of study on the flexural behavior of GFRP tubes filled with magnetically driven concrete.

In this study, concrete-filled GFRP tubes filled with magnetically driven concrete were fabricated. The filled concrete was vibrated by a magnetic vibration device developed by authors. Bending test was conducted to investigate the flexural behavior of the test specimens. In addition, the effect of reinforcing bars was also investigated.

## 2. Experimental Investigation

### 2.1. Magnetically Driven Concrete

In the magnetically driven concrete, the coarse aggregates were totally replaced by steel slag (in weight). The mix proportions of the concrete are presented in Table 1. The cement is Normal P.O Type 42.5 Portland cement. The fine aggregate is natural sand with the fineness modulus of 3.6 and maximum size less than 5 mm. The steel slag was electric furnace slag provide by Harsco Metals Zhejiang Company (Hangzhou, China). Electric furnace slag is able to get larger magnet attraction force since it contains more ferric oxide compared with Converter steel slag. Details of steels slag are presented in Chen et al. [1].

### 2.2. GFRP Tubes

Three series of GFRP tubes, namely series A, B, and C were used in this study. Series A has the nominal diameter of 300 mm and nominal thickness of 12 mm. Series B has the nominal diameter of 300 mm and nominal thickness of 8 mm. Series C has the nominal diameter of 180 mm and nominal thickness of 8 mm. GFRP tubes of Series A and B were fabricated by winding method with the direction of 45°, while the Series C was fabricated by extrusion and winding method with the directions of 45° and 0°.

### 2.3. Reinforcing Bars and GFRP Bars

**Figure 1:**
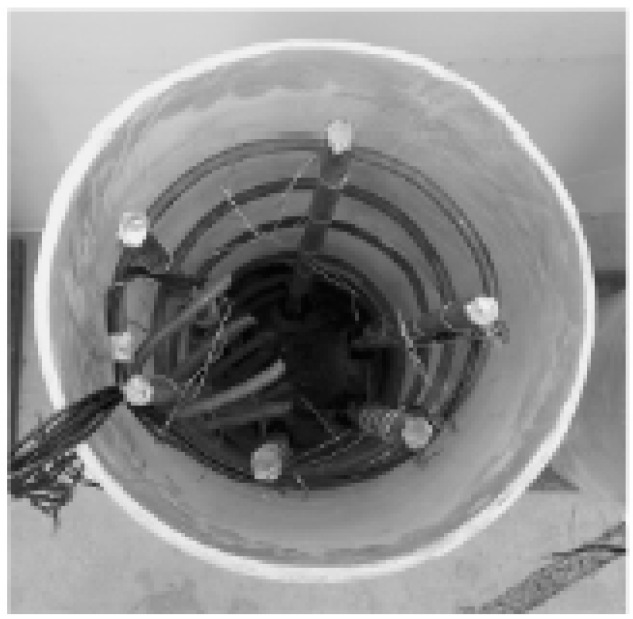
Glass fiber reinforced polymer (GFRP) tubes with GFRP bars.

The reinforcing bars used in this study are Grade HRB335 with the nominal diameter of 6 mm. The nominal diameter of GFRP bars is also 6 mm. The stirrups used are Grade HPB300 with the nominal diameter of 6 mm. The layout of the bars and stirrups is shown in Figure 1. The diameter for stirrups hoop is 100 mm and 200 mm for 180 mm specimens and 300 mm specimens, respectively. The spacing of stirrups is 200 mm for all of the test specimens.

### 2.4. Magnetic Vibration Device and Test Specimens

**Figure 2:**
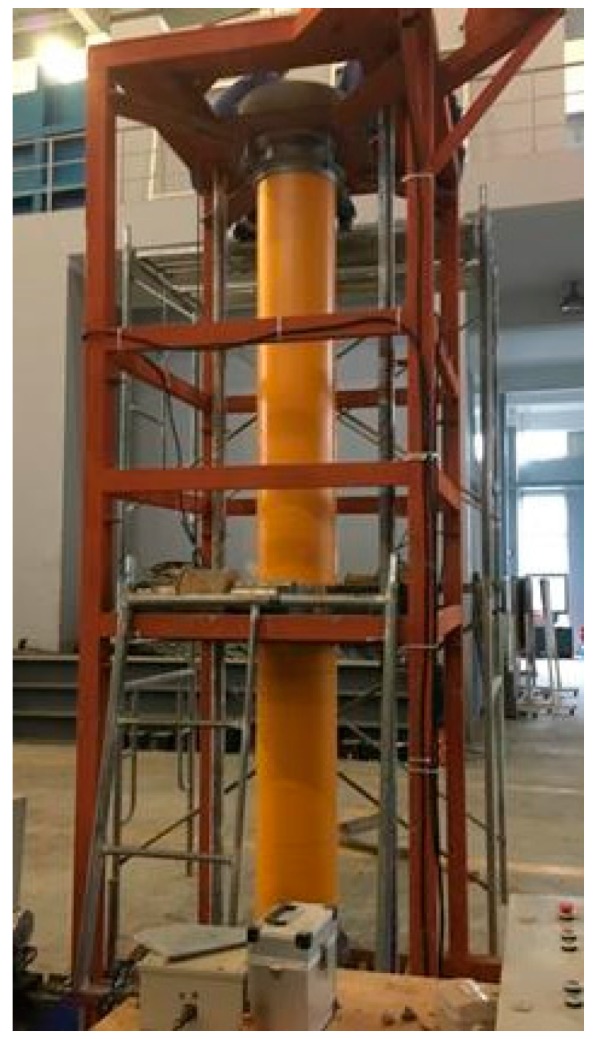
Magnetic vibration device.

A magnetic vibration device was developed for concrete vibration, as shown in Figure 2. The device consisted of a magnetic field generator, rotating base, steel bracket, and control panel. The magnetic field is generated by the four solenoid coils. By switching the direct of electric current on the control panel, the direction of magnetic field could be switched. The GFRP tubes containing concrete were closely surrounded by the four solenoid coil so that the lines of magnetic field were able to pass through the concrete. The coarse aggregates will move under the driven magnetic force. The concrete is vibrated when the direction of magnetic force keeps changing the direction.

The four solenoid coils could move along the length of specimen in order to vibrate the concrete of whole specimen, as shown Figure 2. The required magnetic field for vibration was estimated by using the Maxwell’s equations [14].

In order to vibrate the concrete from 360 degree, the base of the device is able to rotate during the vibrating process with the speed of 30 r/min. In this case, the magnetically driven concrete filled inside the GFRP tube could be vibrated evenly and the coarse aggregates are well-distributed. Each time about 200 mm height concrete were filled and vibrated for about 1.5 min. The magnetic field was controlled reversed at the interval of 0.25 s.

In total, 13 test specimens were fabricated and specimen lengths are 1500 mm and 2000 mm, respectively. Size and number of the specimens are given in Table 2. The test specimens are labeled that the type of bars, length of specimen, diameter of specimen, thickness of GFRP tube, as well as the method of vibration could be identified from the labels.

For example, the label “G2000-300-12A” defines the specimen as follow:

- The first letters indicate that the type of the bars, where the prefix letter “G” refers to GFRP bars while the letter “R” refers to steel reinforcing bars.
- The following four digits “2000” indicate the length of the specimen in mm.
- The following three digits “300” indicate the outer diameter of the specimen in mm.
- The following two digit “12” is the nominal thickness of the GFRP tube in mm.
- The last character “A” refers to the specimen was vibrated using vibrating tube while the character “B” refers to the specimen was vibrated using magnetic method.

### 2.5. Bending Test

**Figure 3:**
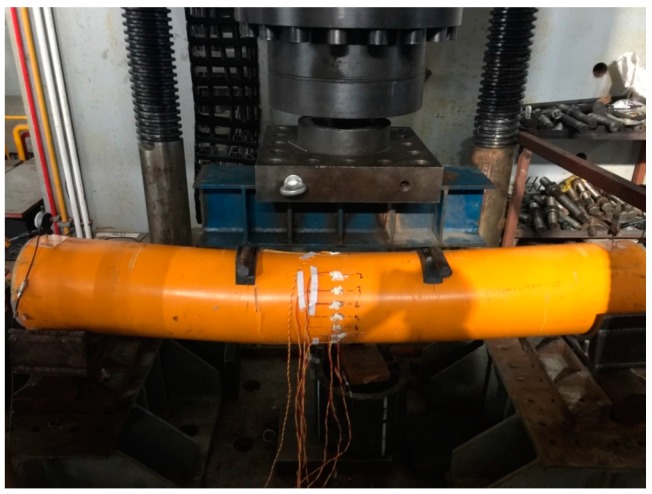
Bending test setup.

**Figure 4:**
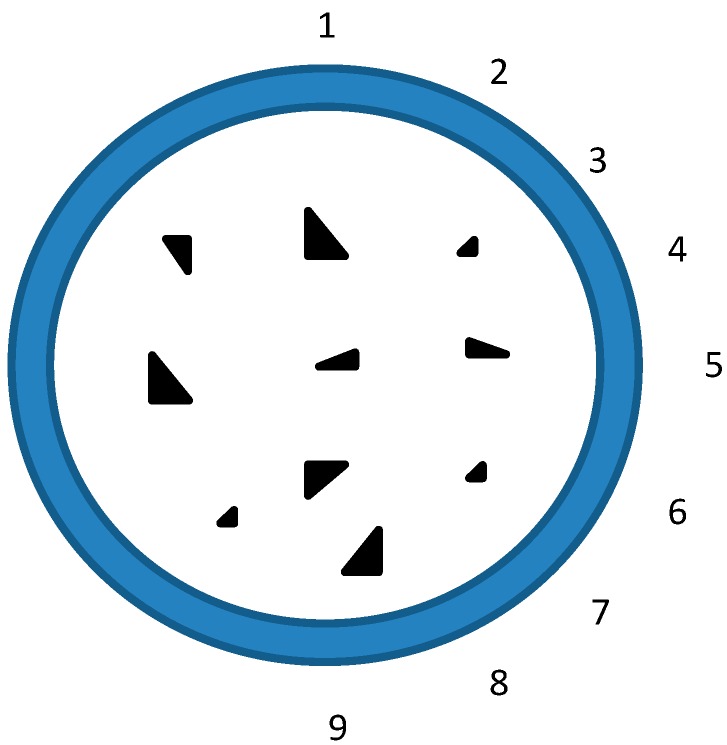
Arrangement of strain gauges.

Four points bending test was conducted and the test specimens were loaded by means of a spreader. Each specimen was simply supported with a 100 mm extent portion overhang at each edge support. Some unloading–reloading cycles with small amplitude of load was initially applied in the elastic domain. Then, the load was slowly applied at the constant speed of 1.0 mm/min. A 10,000 kN YAW-10000F hydraulic testing machine (HuaWei, Hangzhou, China) was used to apply the load with displacement control, which allowed for the tests to be continued to the post-ultimate stage. The test setup is shown in Figure 3. The displacement at the mid-span of the specimen was measured using LVDT while the settlements of the supports were measured by two dial indications. Nine strain gauges were attached around the GFRP tubes in the in the direction of specimen length at the mid-span, as shown in Figure 4. The test data of load, displacement, and strain were recorded by a data acquisition system at regular intervals.

## 3. Test Results

### 3.1. Material Test Results

Tensile coupon tests were performed on the same batch of materials to obtain the properties of GFRP used in the test specimens. The measured material properties of GFRP tubes are shown in Table 3. The material properties of the reinforcing bars, GFRP bars, and stirrups were also obtained from the tensile test, as shown in Table 4. The compressive strength (*f_cu_*) obtained on 150 mm cubic specimens at 28 days for concrete vibrated by magnetic method and vibrating tube were 44 MP and 46 MPa, respectively. Concrete cubic specimens used the same materials as the beam specimens. The compressive test of concrete cubic followed the Chinese Standard [15]. Compressive tests were carried out on 28-day concrete cubic specimens cured in standard environment specified in Chinese Standard [15]. The compressive strengths were the average value of three concrete cubic specimens.

### 3.2. Beam Test Results

#### 3.2.1. Load-Displacement Curves

**Figure 5:**
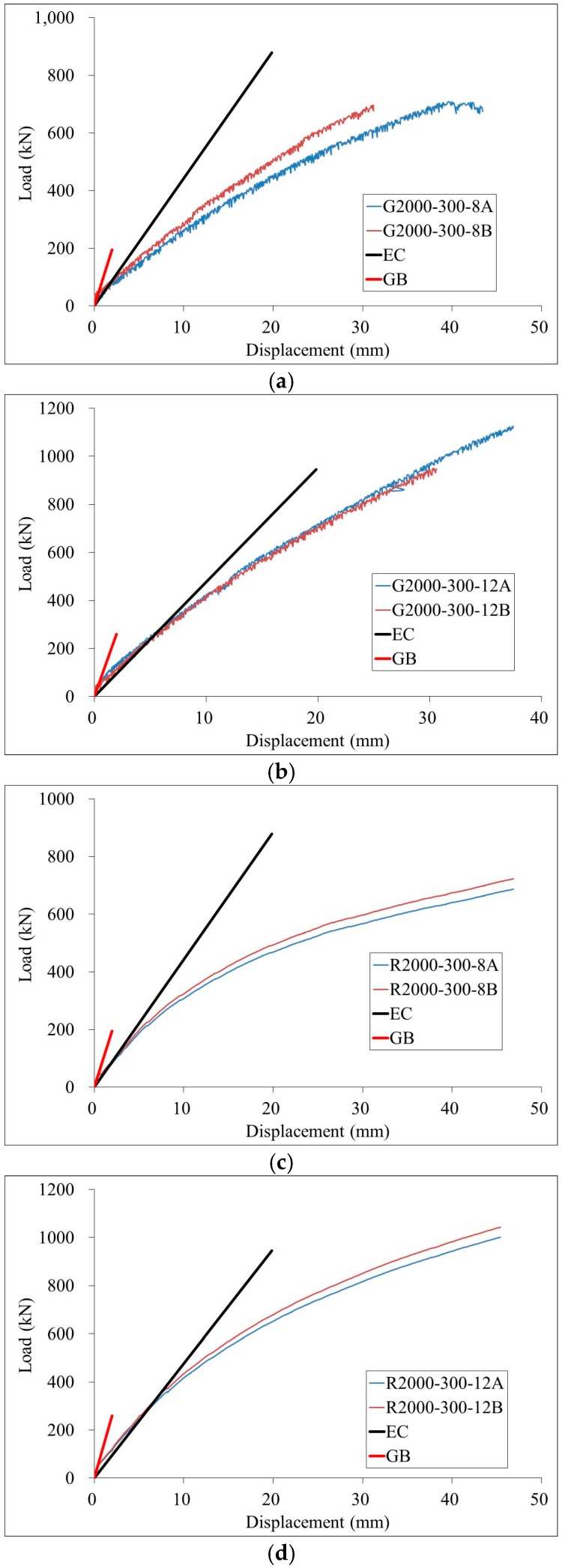

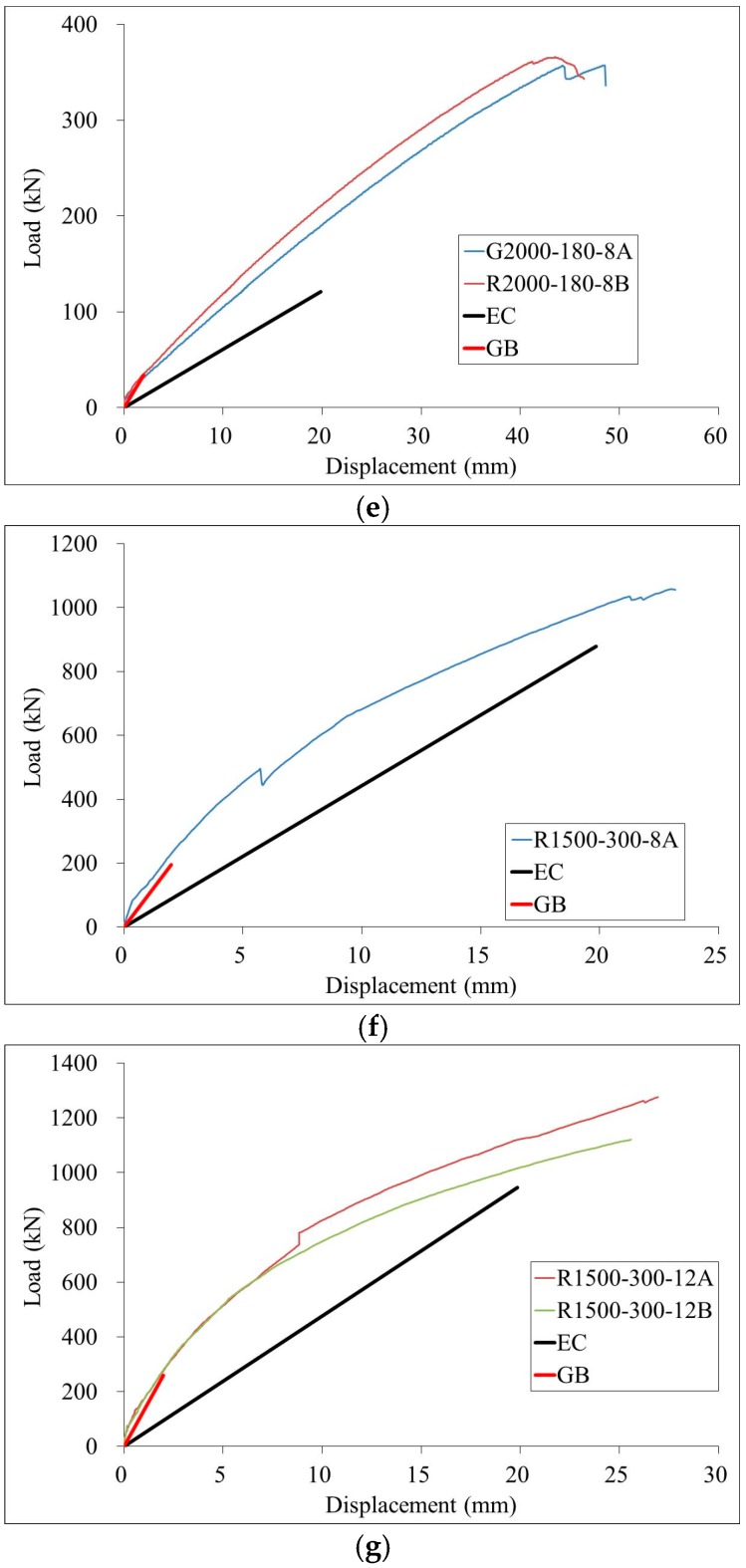
Load-displacement curves of test specimens and predictions (**a**) Series G2000-300-8 (**b**) Series G2000-300-12 (**c**) Series R2000-300-8 (**d**) Series R2000-300-12 (**e**) Series 2000-180-8 (**f**) Specimen R1500-300-8A (**g**) Series R1500-300-12 (Note: GB is prediction from GB Standard [16] and EC is prediction from Eurocode [17]).

The load-displacement curves at the mid-span of test specimens were presented in Figure 5. It is shown that load-displacement curves could be divided into two stages. In the first stage, the load-displacement curves are approximately linear until the load reaches about 30–40% of ultimate strength. In the second stage, the load-displacement curves exhibit nonlinear behavior. However, the mid-span deflection to span ratio of 2000 mm and 1500 mm specimens beyond 1/70 and 1/60, respectively. Therefore, the ductility of test beams is relatively good. 

Generally, it is shown that the mid-span deflections of specimens vibrated using magnetic method are slightly larger than those specimens vibrated using vibrating tubes. This may mean that the elastic stiffness of concrete vibrated using magnetic method is slightly small than concrete vibrated using vibrating tubes. However, the difference is very small and the effect of magnetic vibration method is acceptable.

**Figure 6:**
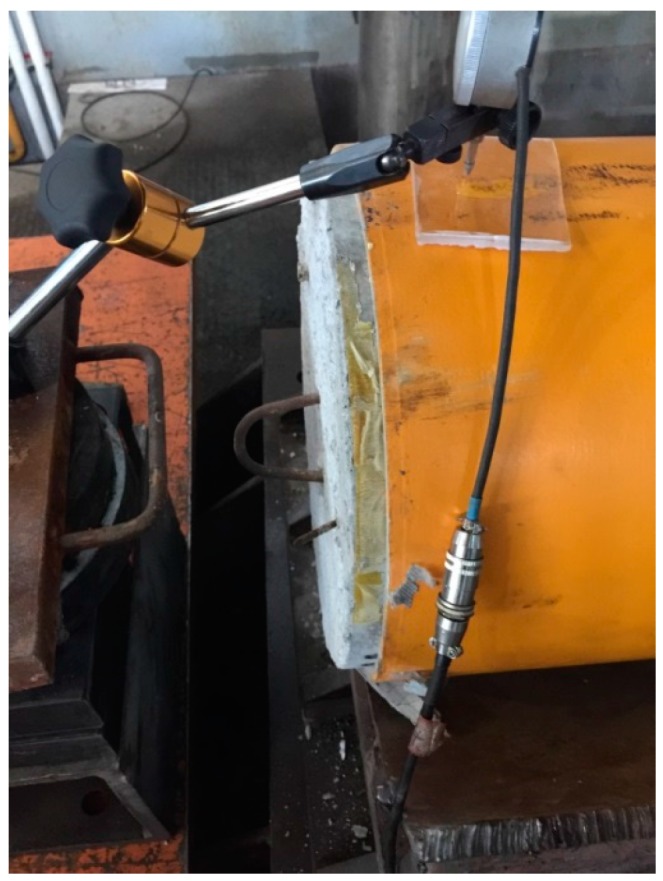
Slip between GFRP tube and concrete.

There is an obvious slip between the GFRP tubes and concrete for specimen under bending, as shown in Figure 6. The difference between the load-displacement curves of specimens having reinforcing bars and GFRP bars is negligible, which may mean that the effect of reinforcing bars on the magnetic field is also negligible.

#### 3.2.2. Load-Strain Curves

**Figure 7:**
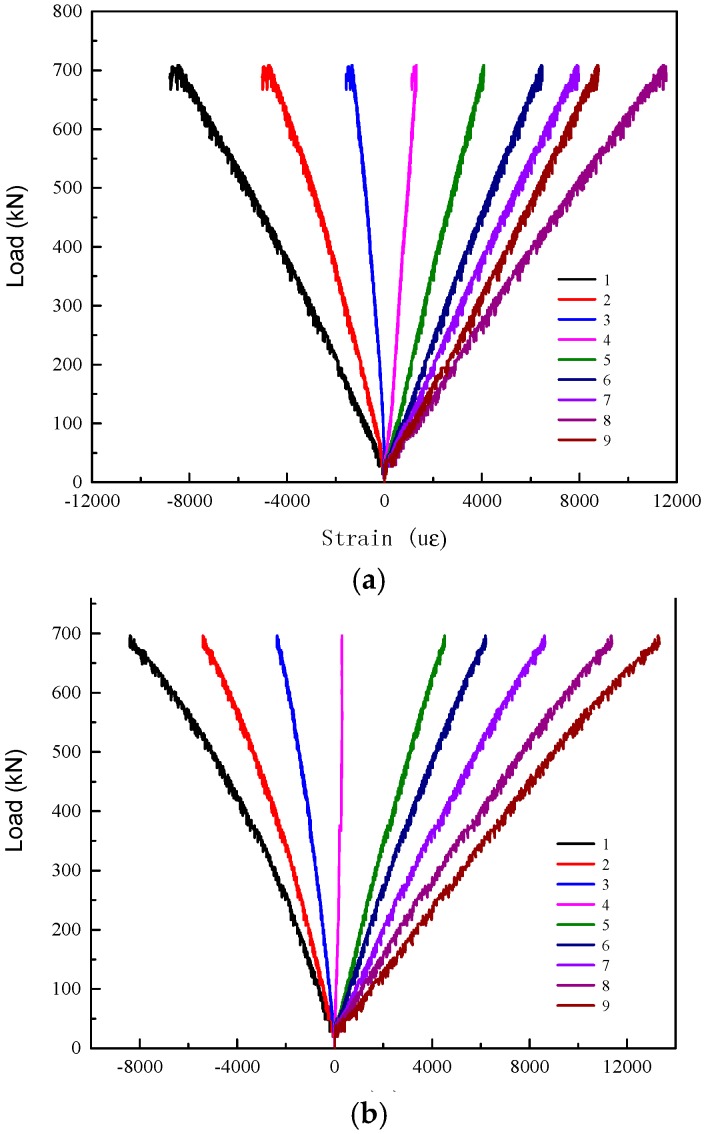
Load-strain curves of specimen series G2000-300-8. (**a**) Specimen G2000-300-8A (**b**) Specimen G2000-300-8B.

**Figure 8:**
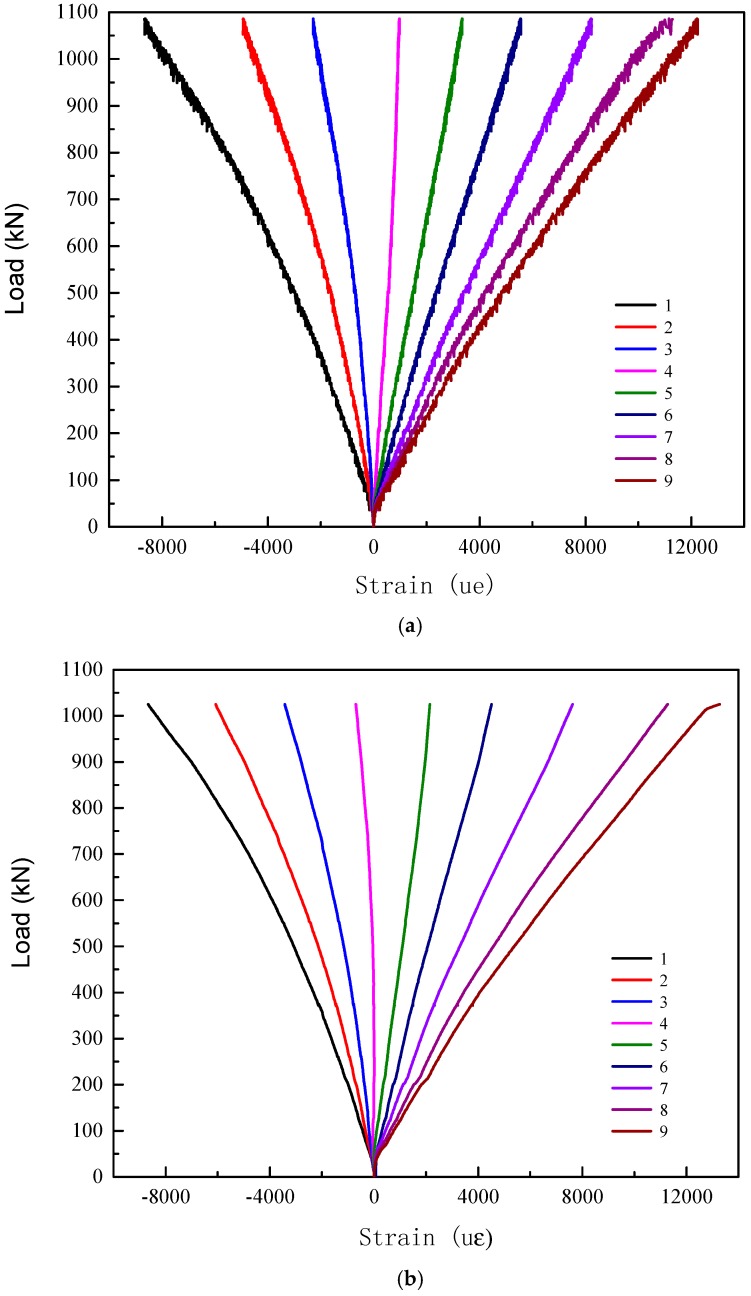
Load-strain curves of specimen series R2000-300-12. (**a**) Specimen R2000-300-12A (**b**) Specimen R2000-300-12B.

Typical load-strain curves of test specimen series G2000-300-8 and R2000-300-12 are shown in Figure 7 and Figure 8, respectively. It is shown that the load-strain curves of each specimen are similar. The mid-span section of GFRP tube remains plane under bending. All of the load-strain curves almost remain linear during the loading process. The values of strain gauge 1 and 2 are almost identical in the elastic stage, which means that there on local buckling occur under in the compression zone. The neutral axis of the mid-span section is at the mid-height initially and gradually moves toward the top of section with the increasing loading.

#### 3.2.3. Failure Mode and Ultimate Strength

**Figure 9:**
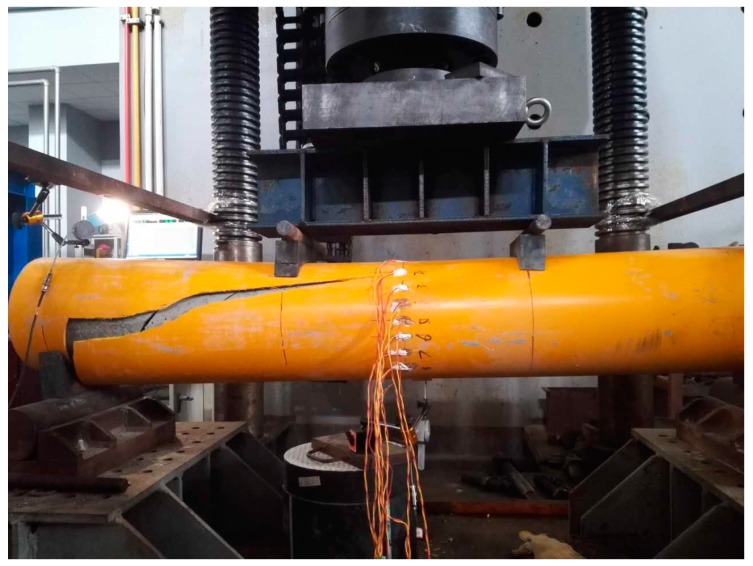
Failure mode of test specimen G-2000-300-8B.

**Figure 10:**
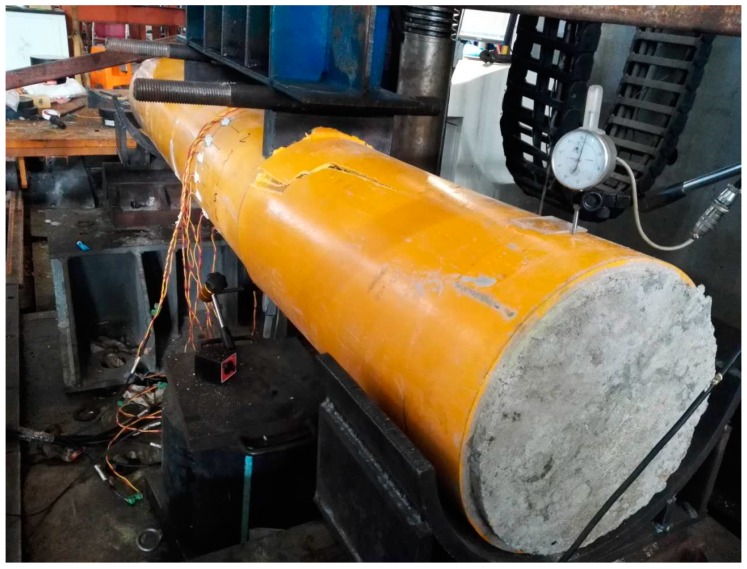
Failure mode of test specimen G-2000-300-12B.

**Figure 11:**
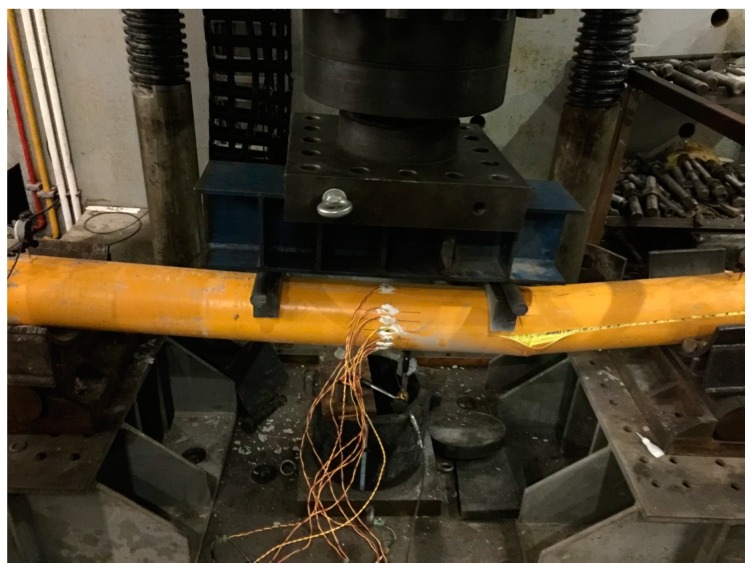
Failure mode of test specimen G-2000-180-8A.

All of the test specimens failed in the crack of outer GFRP tubes and typical failure modes are shown in Figure 9, Figure 10 and Figure 11 for test specimens G-2000-300-8B, G-2000-300-12B, and G-2000-180-8A, respectively. The ultimate strength obtained from test results were presented in Table 5. The ultimate strength of moment could be calculated as *M_u_* = 0.6 *N_u_*/2 and *M_u_* = 0.5 *N_u_*/2 for 2000 mm and 1500 mm specimens, respectively. Specimen G2000-300-12A has the maximum ultimate strength of moment (*M_u_*), while specimen G2000-180-8 has the minimum maximum ultimate strength of moment (*M_u_*). It is shown the 1500 mm specimens have relative large *N_u_* value when compared with 2000 mm specimens having the same cross section, which may due to the failure mode. The comparison between specimen series A and B indicates that the vibration method has a negligible effect on the ultimate strength of test specimens.

## 4. Design of Elastic Stiffness

Two methods were used to calculate the flexural stiffness in this study. The first method is specified in GB 50936 [16] for concrete-filled steel tubes. The second method is specified in Eurocode [17] for effective flexural stiffness for concrete-filled steel tubes having reinforcing bars. The suitability of two methods for concrete-filled GFRP tubes under bending was evaluated in this study. The measured material properties of GFRP tubes were used in the calculation.

### 4.1. Method 1

The flexural stiffness *B_scm_* of concrete-filled steel tubes specified in GB 50936 [16] could be calculated using Equations (1)–(6), the material properties of GFRP tubes were used in the equations:

(1) $$B_{scm}=E_{scm}I_{sc}$$

(2) $$E_{scm}=\frac{\left(1+\delta /n\right)\left(1+\alpha_{sc}\right)}{\left(1+\alpha_{sc}/n\right)\left(1+\delta \right)}E_{sc}$$

(3) $$n=E_{c}/E_{s}$$

(4) $$\delta =I_{s}/I_{c}$$

When there is tensile stress, *I_sc_* could be calculated as Equations (4) and (5)

(5) $$I_{sc}=\left(0.66+0.94\alpha_{sc}\right)\left(I_{s}+I_{c}\right)$$

(6) $$\alpha_{sc}=A_{s}/A_{c}$$

where *A_s_* is the cross section area of GFRP tube (mm^2^); *A_c_* is the cross section area of concrete (mm^2^).

### 4.2. Method 2

The effective flexural stiffness (*EI*)*_eff_* of concrete-filled steel tubes specified in Eurocode [17] could be calculated using Equation (7). The measured material properties of GFRP bars were used in the calculation of G series test specimens.

(7) $${\left(\mathrm{EI}\right)}_{\mathrm{eff}}=E_{s}I_{s}+E_{a}I_{a}+K_{e}E_{\mathrm{cm}}I_{c}$$

The calculated results using Equations (1)–(7) are presented in Table 6. It is shown that the effective flexural stiffness (*EI*)*_eff_* are smaller than the flexural stiffness *B_scm_*, since the confined effect on concrete is not considered in Eurocode [17]. The load versus mid-span deflection curves calculated using the results in Table 6 are compared with the test results in Figure 5. It is shown that the predictions from GB [16] agree with test results well for specimen series 2000-180-8 and specimens having length of 1500 mm. For other test specimens, the predictions from Eurocode agree with the test results well.

## 5. Conclusions

Bending test on GFRP tubes filled with magnetically driven concrete was conducted in this study. Test results of load-displacement curves, load-strain curves, failure mode, and ultimate strengths were obtained. It is shown that the flexural behavior of specimens vibrated using magnetic method and specimens vibrated using vibration tube is similar. The steel reinforcing bars have almost no effect on the magnetic vibration. There is obvious slip between the GFRP tube and concrete at the end of test specimens. Design methods that are specified in current Eurocode and GB for concrete-filled steel tubes were used to calculated the flexural stiffness of test specimens. It is shown that the predictions from GB agree with test results well for specimen series 2000-180-8 and specimens having length of 1500 mm. For other test specimens, the predictions from Eurocode agree with the test results well.

## Figures and Tables

**Table 1:** Mix proportion of the concrete (by weight kg/m^3^).

Water	Cement	Sand	Coarse Steel Slag
210	525	524	1115

**Table 2:** Geometric size of test specimens.

Test Specimen	*L* (mm)	*D* (mm)	*t* (mm)	*d* (mm)	*n* (mm)
G2000-300-8A	2000	300.2	7.98	6	6
G2000-300-8B	1999	300.1	7.98	6	6
G2000-300-12A	2001	299.7	11.98	6	6
G2000-300-12B	2002	300.3	12.01	6	6
R2000-300-8A	2001	300.2	8.01	6	6
R2000-300-8B	1998	300.1	8.01	6	6
R2000-300-12A	2000	300.0	12.01	6	6
R2000-300-12B	2000	299.8	12.02	6	6
G2000-180-8A	2001	180.1	8.00	6	6
R2000-180-8B	2002	180.2	7.98	6	6
R1500-300-8A	1501	300.3	7.99	6	6
R1500-300-12A	1499	300.1	12.02	6	6
R1500-300-12B	1502	300.2	12.03	6	6

Note: *L* is the length of specimen, *D* is the outer diameter of specimen, *t* is the thickness of GFRP tube, *d* is the diameter of bar, and *n* is the number of the bar.

**Table 3:** Material properties of GFRP tubes.

GFRP Tube	Water-Absorption Rate (%)	Degree of Cure (%)	Density (kg/m^3^)	*f_u_* (MPa)	*E* (GPa)
Series A	0.14	92.7	1987.1	371.9	39.2
Series B	0.14	92.5	1985.2	373.1	38.9
Series C	0.15	95.9	2066.5	342.6	36.8

**Table 4:** Material properties of bars and stirrups.

Specimens	*E* (GPa)	*f_y_* (MPa)	*f_u_* (MPa)	*ε_u_* (%)
GFRP bar	31.8	---	519	1.4
Reinforcing bar	205	375	465	---
Stirrups	201	330	415	---

**Table 5:** Ultimate strength of test specimens.

Specimens	*N_u_* (kN)	*M_u_* (kN·m)
G2000-300-8A	708.5	212.6
G2000-300-8B	696.5	209.0
G2000-300-12A	1126.0	337.8
G2000-300-12B	1083.0	324.9
R2000-300-8A	686.9	206.1
R2000-300-8B	723.0	216.9
R2000-300-12A	1001.2	300.4
R2000-300-12B	1043.0	312.9
G2000-180-8A	357.1	107.1
R2000-180-8B	366.0	109.8
R1500-300-8A	1058.0	264.5
R1500-300-12A	1276.1	319.0
R1500-300-12B	1121.1	280.3

**Table 6:** Predictions of flexural stiffness.

Specimens	*B_scm_* (N·mm^2^)	(*EI*)*_eff_* (N·mm^2^)
G2000-300-8A	2.03262 × 10^13^	9.16117 × 10^1^^2^
G2000-300-8B	2.03262 × 10^13^	9.16117 × 10^1^^2^
G2000-300-12A	2.70496 × 10^13^	9.85801 × 10^1^^2^
G2000-300-12B	2.70496 × 10^13^	9.85801 × 10^1^^2^
R2000-300-8A	2.03262 × 10^13^	9.15116 × 10^1^^2^
R2000-300-8B	2.03262 × 10^13^	9.15116 × 10^1^^2^
R2000-300-12A	2.70496 × 10^13^	9.84799 × 10^1^^2^
R2000-300-12B	2.70496 × 10^13^	9.84799 × 10^1^^2^
G2000-180-8A	3.54573 × 10^1^^2^	1.26122 × 10^1^^2^
R2000-180-8B	2.03262 × 10^13^	1.27145 × 10^1^^2^
R1500-300-8A	2.03262 × 10^13^	9.15116 × 10^1^^2^
R1500-300-12A	2.70496 × 10^1^^3^	9.84799 × 10^1^^2^
R1500-300-12B	2.70496 × 10^1^^3^	9.84799 × 10^1^^2^

## Nomenclature

*E_scm_*	the elastic bending modulus of concrete-filled tubes
*I_s_*	the moment of inertia of GFRP tube
*I_c_*	the moment of inertia of concrete
*E_s_*	the elastic modulus of GFRP
*E_c_*	the elastic modulus of concrete
*E_cm_*	secant modulus of elastic of concrete, *E_cm_* = 9500 (*f_ck_* + 8)^1/3^
*I_sc_*	the moment of inertia of concrete-filled tubes
*A_s_*	the cross section area of GFRP tube
*A_c_*	the cross section area of concrete
*K_e_*	0.6 is a correction factor
*E_a_*	the elastic modulus of GFRP tube
*I_a_*	the moment of inertia of GFRP tube
*f_ck_*	the characteristic cylinder compressive strength of the concrete at the age considered
